# The Effect of Temperature, Rest Periods and Ageing on the Response of Bituminous Materials in Fatigue Tests: Considerations and Proposals on Analytical Dimensioning Models

**DOI:** 10.3390/ma15030790

**Published:** 2022-01-21

**Authors:** Félix E. Pérez-Jiménez, Rodrigo Miró, Ramón Botella, Teresa López-Montero, Adriana H. Martínez

**Affiliations:** Department of Civil and Environmental Engineering, Universitat Politècnica de Catalunya, 08034 Barcelona, Spain; edmundo.perez@upc.edu (F.E.P.-J.); r.miro@upc.edu (R.M.); ramon.botella@upc.edu (R.B.); teresa.lopez@upc.edu (T.L.-M.)

**Keywords:** bituminous materials, fatigue tests, temperature, rest periods, ageing

## Abstract

One of the basic assumptions of analytical dimensioning models of asphalt pavements is failure due to fatigue cracking of the bituminous layers. Furthermore, it is considered that the damaging effects of different traffic loads are linear and cumulative, per Miner’s law. However, the analysis carried out on the effect of temperature, rest periods, and ageing of the bituminous materials questions considering fatigue failure as the only and main assumption for the calculation of the pavement life. Ageing of the pavement asphalt layers results in stiffening and transverse cracking. Consequently, these asphalt layers are no longer of infinite extent in the horizontal direction and their response resembles that of a slab. The application of this last calculation assumption provides pavement sections more in line with those used in Spain in the Catalogue of Structural Sections, which is based on experience gained from the real behavior of those sections. The calculation based on the fatigue laws results in undersized structures. This paper shows the results of a strain sweep test implemented at the UPC Roads Laboratory, which was used to analyse all the aforementioned effects and to propose a calculation procedure for heavy traffic pavements considering transverse cracking of the asphalt layers.

## 1. Introduction

Within the analytical pavement dimensioning methods, the most used calculation procedure is based on the assumption that the pavement behaves as a set of superimposed continuous elastic layers, which fail due to fatigue cracking. The calculation method consists of determining the stresses produced by the loads at the base of the bituminous layer of the pavement and estimating the number of cycles that the pavement can withstand as these loads pass, from the fatigue law of the bituminous mixture [1,2].

For the calculation of the pavement stresses, it is assumed that all the pavement layers, including the granular layers and subgrade, are elastic and can be characterised by their modulus of elasticity and Poisson’s ratio. The response of these materials, and that of the bituminous mixtures in a very particular way, is very much affected by the load application speed. For this reason, cyclic tests are used in the characterisation of bituminous mixes, which apply stresses at high frequencies (10–30 Hz) or load pulses at high speed [3,4].

Cyclic loading is also the basis of the tests for the fatigue analysis of bituminous mixes, since they allow the application of a large number of loads in a very short time.

In the determination of the fatigue laws of the bituminous mixes, a flexural test is usually performed, which consists of subjecting a beam to a repeated flexural bending at a controlled strain amplitude [5].

**Figure 1:**
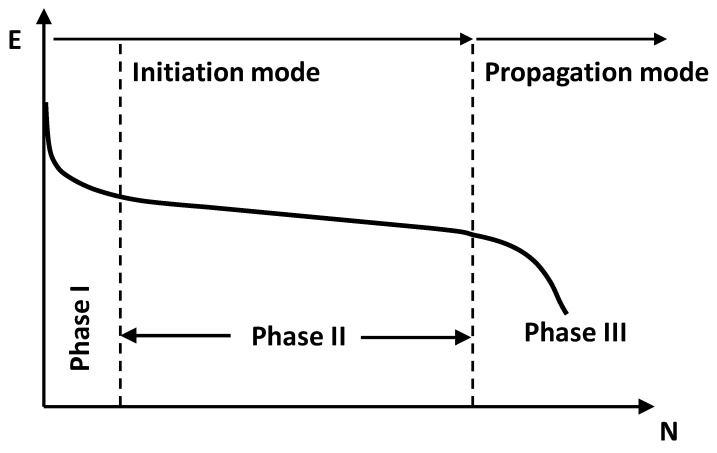
Fatigue graph with the phases.

The evolution of the modulus with the number of cycles is obtained in the test. This curve shows three areas that were interpreted in the following way (Figure 1 [6]):

Phase I: Sharp drop of the applied load. This is associated with different phenomena, principal among which are the adaptation of the specimen to the test and the non-linearity of the material.

Phase II: Practically linear loss of resistance. Part of the test associated with fatigue failure.

Phase III: Rapid drop of the load at the end of the test (failure phase).

Sometimes, due to the ductile response of bituminous mixes, this third phase does not appear. Therefore, mixtures are usually considered to have failed when the applied load is reduced to 50% of that applied in the initial cycles, cycle 100 or 500, in accordance with the standardized procedures. The modulus determined in this cycle is considered representative of this mechanical characteristic of the tested mixture.

All these guidelines and rules are considered to liken the response of bituminous mixes in fatigue tests to that of metal or concrete, where a clear crack is usually observed, which is not appreciated in the majority of bituminous mix specimens when the test is carried out at intermediate temperatures, between 10 and 20 °C.

This adaptation of the behaviour of the bituminous materials to the response of an elastic material with continuous deterioration during the loading process can be seen in the procedure followed in the application of the viscoelastic continuum damage (VECD) model [7,8], in the analysis of the LAS test results [9], and in the determination of the number of cycles that cause the fatigue failure of these materials.

In this procedure, the Schapery Equation (1), which relates the potential work applied to a material (*W*), the energy used for its strain, and its damage (*D*), was modified (2) in order to eliminate the energy associated with the viscous behaviour of the material from the potential energy (the hysteresis loop), through the use of the pseudo-strain $\left(\gamma_{i}^{R}\right)$ (3) and pseudo-energy (*W^R^*) (4).

(1) $$\frac{dD}{dt}=-\frac{dW}{dD}$$

(2) $$\frac{dD}{dt}={\left(-\frac{dW^{R}}{dD}\right)}^{\alpha }$$

(3) $$\gamma_{i}^{R}=G^{*}\cdot \gamma_{i}\cdot sen\;\left(w\cdot t+\delta \right)$$

(4) $$W_{i}^{R}=\frac{1}{2}\cdot \tau_{i}\cdot \gamma_{i}^{R}=\frac{1}{2}\cdot C_{i}\cdot {\left(\gamma_{i}^{R}\right)}^{2}$$

The pseudo-strain comes from the strain measured in each cycle, to which the initial phase angle has been added, so that the hysteresis loop and the pseudo-energy are practically nil at the start of the cyclic tests.

The damage intensity *D*(*t*) is considered in the VECD model and in the LAS test as the sum of the energies dissipated in each cycle.

(5) $$D\left(t\right)≅\overset{N}{\underset{i=1}{\sum }}{\left[\frac{1}{2}\cdot {\left(\gamma_{i}^{R}\right)}^{2}\cdot \left(C_{i-1}-C_{i}\right)\right]}^{\frac{\alpha }{1+\alpha }}{\left(t_{i}-t_{i-1}\right)}^{\frac{1}{1+\alpha }}$$

where *C_i_* is the material integrity in each cycle and *α* is a material dependant constant related to the maximum slope m of the master curve that relates the logarithm of complex modulus and frequency.

(6) $$C_{i}=\frac{\tau_{i}}{\gamma_{i}^{R}}$$

(7) α=1+1m

The damage intensity keeps relating to the hysteresis loop, the area of which increases as does the phase angle as the test proceeds. The viscous response of the material is considered as damage, which does not always entail a micro-crack nor deterioration or damage of the material, especially at small strains or stresses.

The advantage of the LAS test and the VECD method for processing data is that the number of cycles to failure can be estimated according to the applied strain by using the DSR with a quick and simple strain sweep test.

(8) $$N=\frac{f\cdot D_{f}^{K}}{K\cdot {\left(\frac{1}{2}\cdot C_{1}\cdot C_{2}\right)}^{\alpha }}\cdot G^{*-2\alpha }\cdot \gamma^{-2\alpha }$$

where *D_f_* is the damage intensity at failure, which corresponds to the maximum stress cycle in the LAS test. *C*_1_ and *C*_2_ are coefficients of curve fitting.

In the case of bituminous mixes, flexural cyclic tests with prismatic specimens are usually performed at different strain levels to obtain the fatigue law. This test is long and costly. Therefore, the objective of this study is to propose a quicker test that was used to analyse the effects of temperature, rest periods and ageing. The UPC Roads Laboratory implemented a strain sweep tension-compression cyclic test, called EBADE test (from the Spanish acronym of ensayo de barrido de deformaciones). This test can be applied both to bitumens and bituminous mixes, and allows the fatigue law to be estimated based on the determination of the strain level that produces the rapid failure and the strain level at which no deterioration occurs.

In contrast to the LAS test, in which the strain increase process is carried out quickly and continuously, in the EBADE test, the strain increase is discontinuous and 5000 cycles are carried out at each strain level.

In both tests, the material failure and the fatigue laws correspond to the deterioration that occurs on applying a continuous series of cyclic tests, but the cause of this failure and the effect that the rest periods have are not known. Consideration of these factors is very important because Miner’s law is also used in the application of the fatigue laws to the analysis of pavements, which assumes a continuous and linear damage process.

The application of Miner’s law is clear in the case of elastic and brittle materials in which the loss of stiffness is associated with microcracks, as with concrete, but not in the case of bituminous materials in which this loss can be associated with a thixotropic behaviour [10]. In addition, the ageing effect and the temperature should also be taken into account in the response of the bituminous materials, which can change from ductile and viscous to stiff and brittle.

In the fatigue failure calculation for bituminous pavements, Miner’s law is also applied, which assumes that the damage produced by the loading is linear and accumulative. This allows the effect of different types of loading and that of the ambient temperatures and conditions to be taken into consideration.

However, upon further analysis of the mixture response in fatigue tests, it is questioned whether these calculation assumptions can always be applied and whether fatigue failure is the only deterioration mechanism to be considered.

This analysis of the damage process, or more accurately, of the change of behaviour of the bituminous materials under the application of cyclic loading, has been carried out by means of EBADE test. Thus, the change of response of the material can be seen at the same strain level as well as the result of the strain increase.

This test was used to analyse:

- The relationship between the dissipated energy and the evolution of the rheological properties of the bitumens and bituminous mixes under cyclic loading.
- The effect of the applied strain level and the repetition of cycles at each level.
- When and how bituminous materials recover their initial properties as the applied strain reduces or the test is stopped.
- The effect of the rest periods on the resistance to fatigue failure. The application of Miner’s law.
- The change of behaviour due to the effect of temperature (ductile or brittle response).
- The effect of ageing.

Also considered is how all these effects can influence the application of the fatigue laws obtained in the standardised tests in the pavement design. Lastly, a new calculation procedure is presented for heavy traffic pavements, which considers transverse cracking of the asphalt layers as a consequence of their ageing.

The paper is a synthesis of the work carried out for years in the application of the EBADE test, which shows how the characterization of bituminous materials is easier and more complete if it is based on the thixotropic response than by associating it with a visco- elastic model as is usually done. In addition, the different hypotheses normally used in pavement design, e.g., multilayer structures, continuity of layers, Miner’s law, and healing, should be reviewed on the basis of the results obtained in these tests.

## 2. EBADE Test

The EBADE test is a strain sweep, tension-compression cyclic test, which is applied to bitumens, mastics, and bituminous mixes for the analysis of the behaviour of these materials under repeated cyclic loading. This strain sweep is carried out by increasing the imposed strain level every 5000 cycles. The test can be carried out at different temperatures with the aim of observing its effect on the response of the bituminous materials.

**Figure 2:**
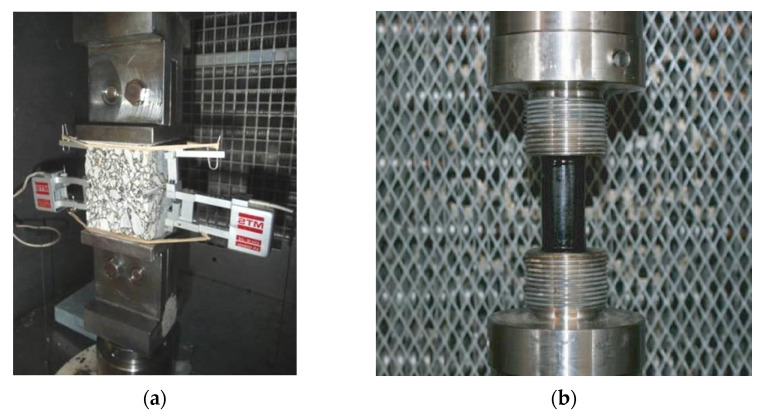
EBADE tests set up for (**a**) prismatic asphalt mixture and (**b**) cylindrical asphalt bitumen specimens.

In the case of bitumens and mastics, the tests are carried out on cylindrical specimens, which are manufactured by pouring the hot material into cylindrical moulds of 20 mm in diameter and 40 mm in height [11]. The initial strain is 0.076%, and the strain is increased by the same quantity every 5000 cycles. For the testing of mixes, a prismatic specimen is used of 6 cm in height with a base of 5 × 5 cm^2^, which is obtained by sawing cylindrical or prismatic specimens, compacted in Marshall moulds or a wheel tracking machine [12]. In this case, the strain amplitude applied to the specimen is +/−25 µm/m at the start, which is increased by the same magnitude every 5000 cycles. The test can be carried out at any temperature, although the normally used range has been between –5 and 20 °C. The tests are carried out at a frequency of 10 Hz on the bitumens, mastics, and mixes, as shown in Figure 2.

The test records the variation of load applied, from which the variation of the maximum stress applied in each cycle and the associated modulus are obtained.

The energy dissipated in each cycle is also determined in this test from the area of the hysteresis loop. This parameter was calculated by Dr. Botella in his doctoral thesis and has been very useful in demonstrating the basis of the change of behaviour of these materials.

When this test is applied at temperatures at which the bituminous materials have a ductile response, between 10 and 20 °C, the evolution of the stress, the complex modulus, and the dissipated energy with the number of cycles has a characteristic response, which is described below.

**Figure 3:**
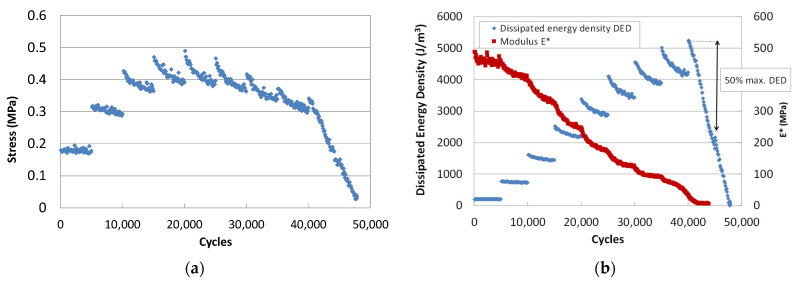
Evolution of (**a**) stress amplitude and (**b**) stiffness modulus and dissipated energy density with loading cycles.

The evolution of the stress decreases within each strain step. During the initial steps, Figure 3a, there is a sharp increase each time the strain level is increased, until a step is reached at which a maximum occurs. Then, it decreases both within the strain step and when the strain level increases. It can also be observed that the breakage is very ductile, i.e., several strain steps can be applied from when the load maximum occurs until failure can be determined [13].

For its part, the modulus shows a continuous decrease, which is more pronounced in the first cycles of the initial steps, as shown in Figure 3b. It then decreases continuously and more slowly, and finally there is a quick drop at the end of the test.

It is difficult to determine failure from the stress or modulus curves; it is easier to define it from the dissipated energy-cycle curves, shown in Figure 3b. The dissipated energy decreases within each step and increases with the strain level, as occurs with the stress. In contrast to stress, when arriving at a high strain level, dissipated energy starts to drop sharply. The failure strain is associated with the load step at which the dissipated energy drops below 50% of the maximum.

## 3. Evolution of the Rheological Characteristics and Their Relationship with the Dissipated Energy Phases I and II

The analysis of the results of the time sweep tests is usually based on the assumption that the tested materials lose their initial stiffness and mechanical characteristics due to a microcrack that, in the end, gives rise to cracking and breakage. In materials, such as metals and concretes, where this failure assumption is met, the material has an elastic response at the start of the test and its modulus remains practically constant. As the number of cycles increases, both modulus and resistance start to reduce until failure by cracking and breakage.

In the case of bituminous materials, the response is different. Firstly, the material experiences a high loss of stiffness at the start of the test, phase I, which continues more slowly as the test progresses, phase II, and which again ends with a rapid and sharp loss of its mechanical capacity, phase III. Only phase II is considered fatigue failure. Phase I is associated with the adaptation of the specimen to the test and to the non-linearity of the material.

However, phase I and II correspond to the same type of response of the material. There is an internal modification process of the properties and rheological characteristics, which takes place from the start of the test and is the same in both phases. The loss of stiffness is related to the dissipated energy in each cycle. For the same strain level, there is a linear relationship between the dissipated energy and the modulus of the material. This relationship is the same at the start of the test, phase I, as when it continues in phase II [14].

**Figure 4:**
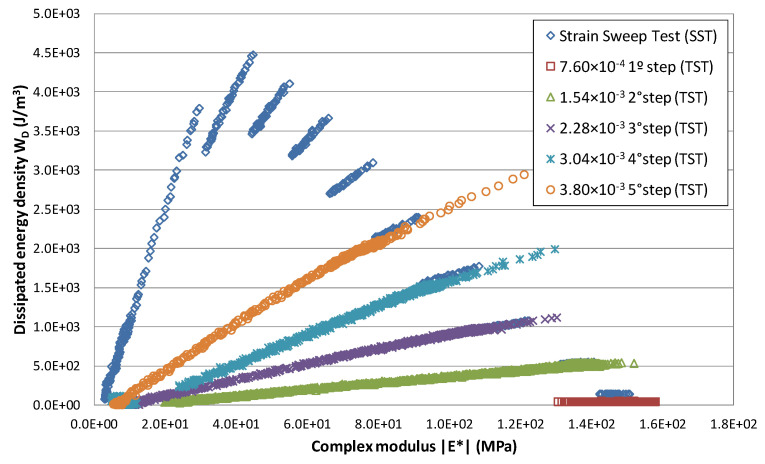
Relationship between the evolution of the modulus and the dissipated energy density in the time and strain sweep tests (60/70 penetration bitumen).

Figure 4 shows the evolution of modulus and dissipated energy obtained when testing a 60/70 penetration bitumen at 10 °C in accordance with the EBADE procedure.

There are straight-line sections drawn with blue rhombuses that move upwards (higher energy) as the strain increases. What in Figure 1 (modulus vs. number of cycles) fits a curve where phase I and phase II can be differentiated, here it becomes a straight line. The variations of the complex modulus are related to the dissipated energy throughout the test.

Also drawn in Figure 4 is the energy–modulus relationship for the time sweep tests that were carried out with the same bitumen at the same strain levels used in the EBADE test, which are the other lines included in the figure (indicated with TST in the legend). All these, except the one in which the lowest strain has been applied, show a significant loss of their modulus at the end, which would indicate their fatigue failure. Only at the lowest strain level has failure not occurred, and the variation of the dissipated energy in this case is nil or very small.

Furthermore, the results obtained in the EBADE are superimposed on those of the time sweep test for the same strain level. The response of the material is mainly related to the imposed strain level and is not affected by the path followed to arrive at this strain level (applied from the start in the time sweep test and by steps of 5000 cycles in the EBADE test).

### Modulus-Phase Angle Curve: Effect of the Strain Level and the Number of Applications

The EBADE test allows a characteristic curve of the bitumen to be obtained, which indicates how it is behaving and responding at any time to the applied strain and how and when failure occurs. Similar curves were shown by other researchers who have studied reversible phenomena during cycling loading on bitumens [15,16].

In viscous materials, the dissipated energy (*W_i_*) is related, in each cycle, to its stress state and to the phase angle by the following expression:

(9) $$W_{i}=\pi \cdot E_{i}{\left(\varepsilon_{i}\right)}^{2}\cdot sen\delta_{i}$$

where

*E_i_*: complex modulus
*ε_i_*: strain
*δ_i_*: phase angle

**Figure 5:**
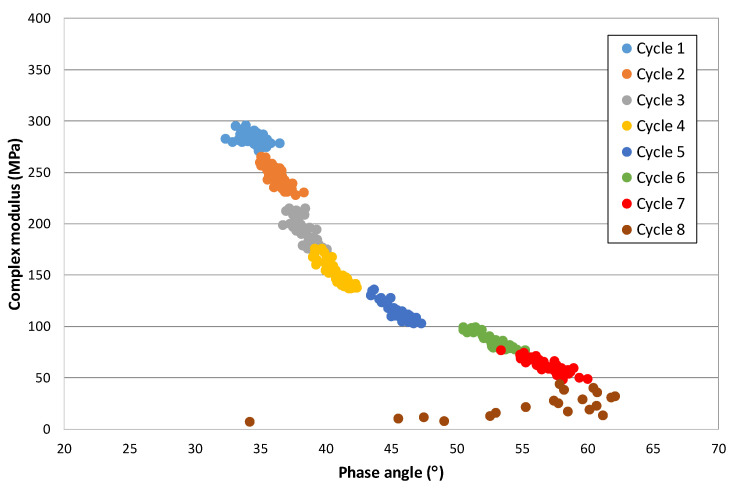
Variation of the modulus with the phase angle at 10 °C (50/70 penetration bitumen).

From this formula, the relationship has been obtained between the complex modulus and the phase angle for each cycle at each strain level. The graphical representation provides another view of the response of these materials. Figure 5 shows this relationship for a 50/70 penetration bitumen at 10 °C (the different strain levels are plotted in different colours). This curve that, in principle, could be considered a Black diagram has been divided into sections and thus shows the response of the bitumen as the strain and the number of cycles increase.

This figure shows that the variation of modulus and phase angle with increasing strain is not linear. At the lowest strain level, the modulus does not vary, but the angle does. As the strain level increases, the modulus decreases significantly, and the phase angle increases. However, there are certain strain levels at which an equilibrium state is reached, and the bitumen maintains its rheological constants with the number of cycles. These strain levels could correspond to the maximum no-damage level, at which point the bitumen would recover its initial properties when the test is stopped. Finally, as the strain continues to increase, there is a further increase in modulus until failure is reached.

## 4. Effect of Unloading Processes and Rest Periods

The loss of modulus observed in the bitumen in the above tests is basically related to the applied strain, while the effect of the loading history is barely considered. This fact led us to analyse whether, as the bitumen specimen was unloaded in a reverse process, it could recover its initial response.

The influence of rest periods on bituminous materials has also been studied with other tests, such as dynamic rheometers or uniaxial tension-compression tests [15,16,17,18,19].

**Figure 6:**
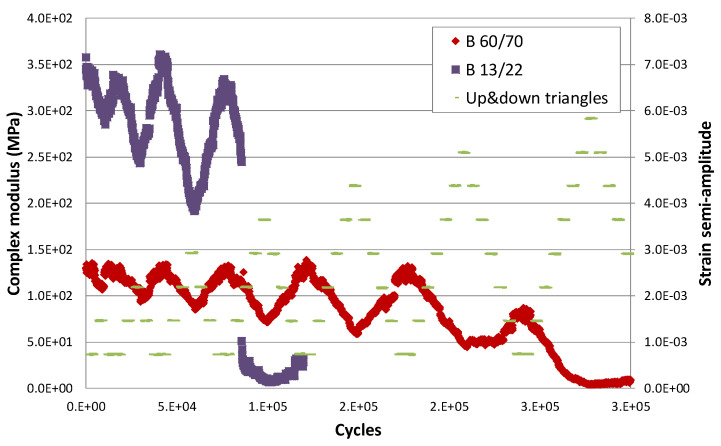
Complex modulus evolution with the number of cycles at 10 °C.

Figure 6 shows the results of applying EBADE test at 10 °C on two bitumens with different penetrations, one harder B13/22 and another softer B60/70 [20]. Table 1 collects the main properties of both bitumens.

In this case, increasing and decreasing strain steps were applied, and the strain level of the final step was progressively increased. Thus, 5000 load cycles were applied to each rising and falling step. The applied strain steps are shown in green on the graph (indicated as up and down triangles).

In the case of bitumen B60/70, the red curve, a higher number of ascending and descending step blocks were able to be applied, namely seven blocks. A higher strain level was reached. In the first five blocks, the bitumen recovers its initial stiffness when the application of the series of descending strains is stopped. Its stiffness is the same as it had at the start at the lowest strain level. That is, there was no damage, and its response was that of a thixotropic material: modulus and load increase as more cycles are applied (thixotropic behaviour).

This response is very different to that of a linear viscoelastic material, where on reducing the strain, the applied load descends slowly, depending on the viscosity, and the modulus remains the same.

In the sixth block, which has a higher final strain level, the material does not recover its stiffness as the strain level reduces; its behaviour has been affected.

In the case of the harder bitumen, B13/22, this failure occurs earlier in the fourth block. Although a certain amount of damage after the application of the third block is now seen, the bitumen does not recover its initial modulus. In the previous blocks, the first and second, this damage is not observed.

There is a range of strains, the softer the bitumen, in which the applied stresses produce a loss of stiffness but do not result in damage. They are a consequence of the thixotropic response of the bitumen. This rheological behaviour has given rise to two misinterpretations when analysed from the point of view of fatigue failure. The first associates the loss of stiffness to a damage process similar to that occurring in concrete and other materials by microcracking, whereas the second assumes that there could have been a later healing process.

### 4.1. Relationship between the Dissipated Energy and Variation of the Internal Temperature

**Figure 7:**
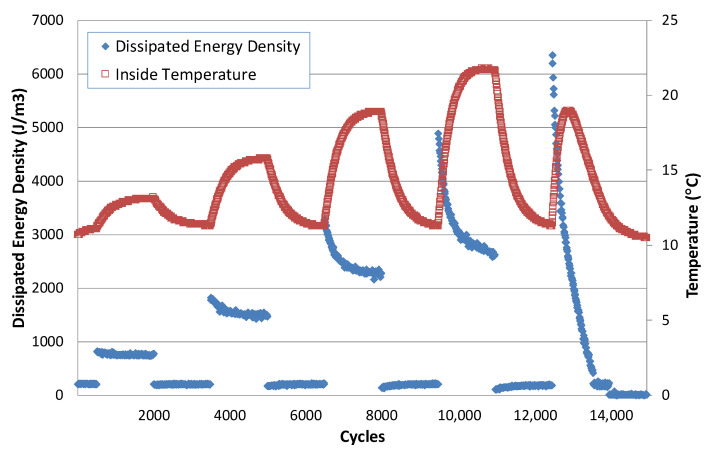
Evolution of dissipated energy density and temperature with number of cycles. EBADE test carried out at 10 °C and 10 Hz.

Another relationship also demonstrated by this test is the one between the dissipated energy and the temperature variation experienced by the bitumen throughout the cyclic tests. Starting from the EBADE test carried out at 10 °C, the temperature variation inside the specimen was measured as the strain steps rose, and steps were interspersed once the initial strain was applied, as shown in Figure 7 [21]. This figure shows the variation of the dissipated energy: it increases with the applied strain level, but it reduces within each step with the number of cycles. When moving to the lowest strain interspersed step, the dissipated energy also goes down again.

The variation of the temperature inside the sample is in line with the dissipated energy. As the strain step rises, the temperature starts to increase until it stabilises at the end of the step. The temperature increases are higher the greater the strain levels. When the lower strain step is applied, the temperature drops, and it goes back to the initial value of 10 °C. The temperature increase with the strain is very significant, up to 13 °C.

In the last step, the dissipated energy reaches a maximum and drops rapidly, while the temperature increase is lower and even starts to drop within the same cycle. The bitumen starts to behave differently. This change of behaviour is associated with failure. This temperature change could influence or be related to the thixotropic response of the bitumens.

### 4.2. Rest Periods and Miner’s Law

The way that the loads are applied, continuously or with rest periods, also has a high influence on the results of the fatigue tests.

**Figure 8:**
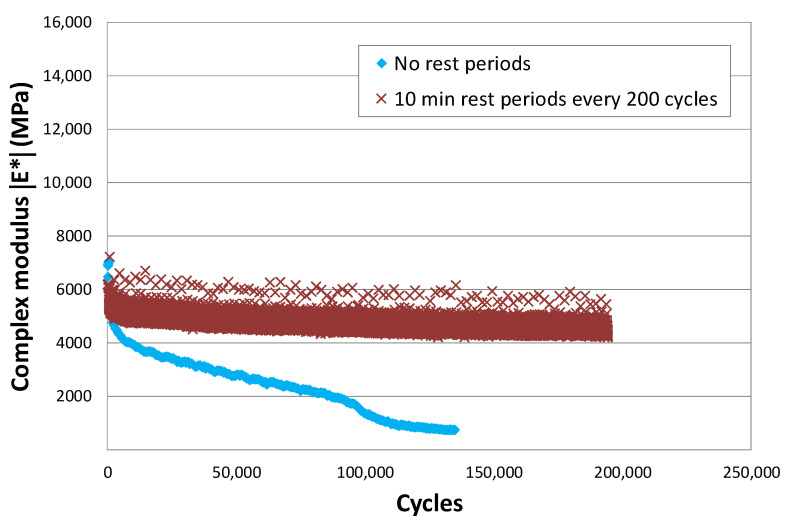
Comparison between the complex modulus with and without rest periods. BBTM mixture with 50/70 binder at 20 °C and 10 Hz. Strain amplitude of 200 mm/m.

Figure 8 shows two tension-compression fatigue tests at the same strain level and at the same temperature, 20 °C [22]. In one, represented in blue, there are no rest periods and in the other, in red, a rest period of 10 min is included every 200 cycles. In the first case, failure occurs before 100,000 cycles are applied, whereas in the other, adding the applied cycles, more than 190,000 load applications are reached with hardly any damage observed. In this last case, the mix continues to give its initial response.

The load application sequence should be considered when applying the fatigue laws of the bituminous materials. These have been obtained in tests where the load is applied very quickly and continuously but, in reality, the load applications on the pavement are more separated in the majority of cases.

The results of this test also bring into question the application of Miner’s law to these materials. This law assumes that the damage produced by a load is continuous and linear and that the damage produced by different loads is accumulative. In this test, we can check how the same load gives rise to different damage levels just by changing the rest periods during their application. In one case, when the loading process is continuous, the damage could be considered linear and accumulative, and in the other, not.

## 5. Effect of Temperature

The ductile and thixotropic response of the bituminous products disappears if the mix or bitumen becomes a stiff and brittle material when the temperature drops. In this condition, the response of the material starts to resemble that of other construction materials, such as concretes, metals, or glasses.

**Figure 9:**
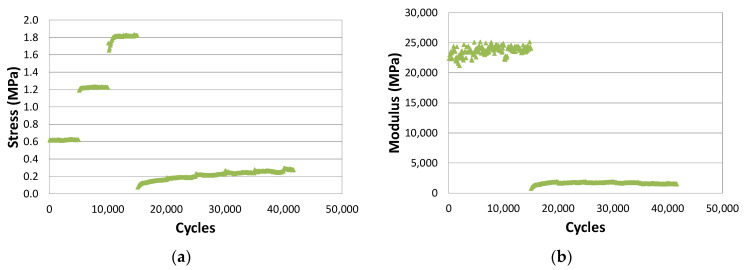
EBADE test results at −5 °C and 10 Hz. (**a**) Stress and (**b**) modulus evolution with number of cycles. AC16 50/70 S mixture.

Figure 9a,b show the results of the EBADE test at −5 °C on a dense bituminous mix specimen manufactured with 5% of a 50/70 penetration bitumen [23]. In the figures, a practically linear relationship can be observed between the stress and the imposed strain level. The modulus hardly changes, neither within the step nor when the step level is increased.

The mixture behaves like an elastic material. Its failure occurs abruptly as the strain level increases and it arrives at the failure strain.

**Figure 10:**
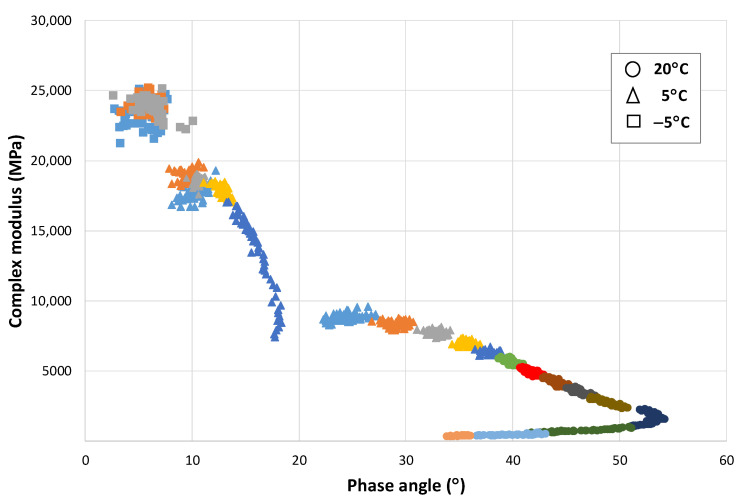
Variation of the modulus with the phase angle. AC16 50/70 S mixture.

This change of behaviour with temperature can be better observed on the modulus-phase angle curve of that mix obtained from the EBADE test carried out at three temperatures, i.e., 20, 5, and −5 °C, as shown in Figure 10. At 20 °C, the response is ductile and the behaviour predominantly thixotropic as previously mentioned. As the strain level increases, the modulus decreases and the phase angle increases. The variation of these parameters differs with the strain level.

At the lowest strain levels, a greater increase of the phase angle is observed while the modulus hardly varies, especially within each strain step. As the strain level increases, both fall more quickly, until reaching the limit value of the phase angle at which a rapid drop of the modulus occurs. The material is not now capable of adapting to the new stress, its phase angle increases, and it loses all resistance and fails.

At low temperatures, −5 °C, the mix has a brittle and linear elastic response. The material maintains its modulus and phase angle during the test. The former reaches very high values, around 25,000 MPa, similar to that of concrete, and its phase angle is very low (around 5°). Failure is also similar to that of concretes and brittle materials, it occurs quickly when the failure strain is reached, like Figure 9a.

At the intermediate temperature, 5 °C, the response of the mix starts to be a little ductile, its failure takes place at a low strain, and the phase angle also moves in low ranges, between 8 and 15°.

## 6. Effect of Ageing

**Figure 11:**
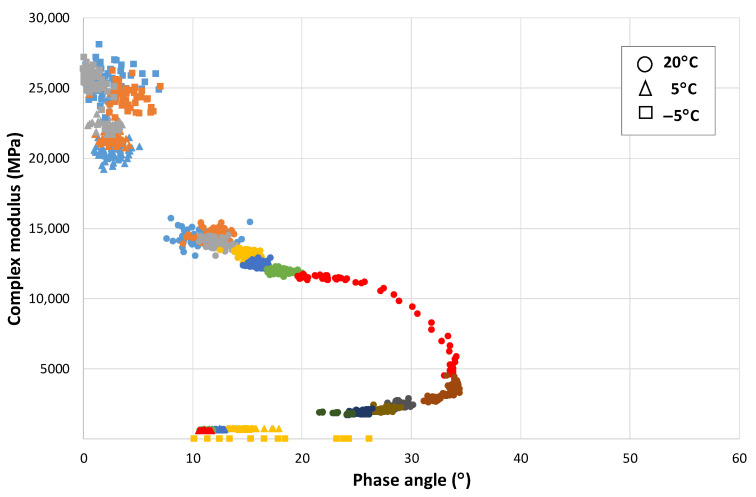
Variation of the modulus with the phase angle. Aged AC16 50/70 S mixture.

As bituminous materials age, they lose their ductility and become stiffer and brittle materials. This can be easily appreciated if we compare the modulus-phase angle curves obtained for the same mix in the EBADE test at 20, 5, and −5 °C, before and after its ageing. In the previous section, Figure 10 shows this curve for an unaged mix. Figure 11 shows this curve for the aged mix.

It can be seen how the mix has increased the stiffness at the three test temperatures and how the range of the phase angle has reduced. At 20 °C, it can be seen how the mix failure takes place at a much lower strain, the seventh step for the aged mix and eleventh for the unaged, with a lower failure phase angle as well, 35 and 55°, respectively.

At 5 °C, it now has a practically elastic response, with a high modulus, 20,000 MPa, and breaks on the third step. It has a similar response to the unaged mix at −5 °C, i.e., the ageing means that the mix has a brittle response at a temperature of 10 °C, higher than that corresponding to a recently manufactured mix.

## 7. Considerations and Proposal

As a summary of that discussed above on the use of cyclic tests for the assessment of fatigue failure of the bituminous materials, first the state of the bituminous material must be considered, whether it is brittle or ductile. In the first case, we can talk about failure by cracking, usual in stiff materials, such as concretes and metals. Failure can be frequently observed in the case of the bituminous layers of the pavements that are not very thick, less than 14–16 cm as a whole, and that at certain times of the year are subjected to low temperatures, that is, when there is a very stiff layer of relatively low thickness on an untreated granular subbase. In this case, a cracking and breakage process occurs that goes from the bottom up.

When the mix has a ductile response, the failure is due to inconsistency. Each load application affects and changes the state of the material, making it even more flexible and ductile. Its failure occurs, as the tests show, after a continuous and very long loading process, unless the mix layer was subjected to a high strain. If the strain is not very high, the number of load applications would still increase if the rest periods can be considered.

In principle, this leads to considering that the failure of flexible pavements by cracking is unlikely when the mix is in the ductile state, unless the thickness of the asphalt layer is very thin and the traffic loadings give rise to a very high strain level at the base of the layer, something that also occurs when the mix layer rests on very low-capacity substrates.

However, the mix is subjected to an ageing process during the service life of the pavement. This ageing means that the response of the mix passes from the ductile to the brittle state. The first consideration to bear in mind would be to use the fatigue laws of the aged mixtures as well in the dimensioning methods. This has been studied in some works and a doctoral thesis carried out in the UPC Road Laboratory.

### 7.1. Effect of Ageing and Temperature on the Calculation of the Fatigue Life

**Figure 12:**
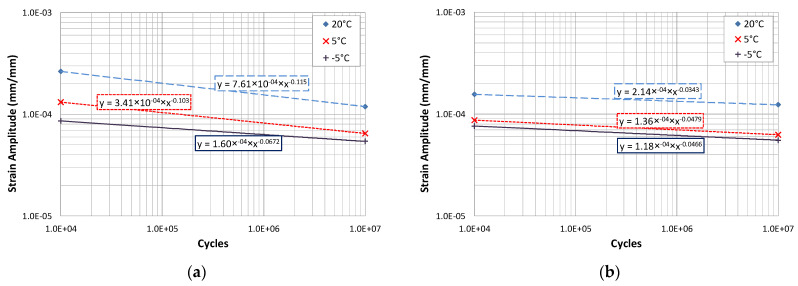
Fatigue law for (**a**) unaged mixture, (**b**) aged mixture.

Table 2 includes the results of applying the fatigue laws for the same bituminous mix according to its state of ageing: recently manufactured and aged (by subjecting the mix to a long ageing process before manufacturing the test specimens). For these two ageing conditions, the fatigue laws were obtained at three temperatures, −5, 5, and 20 °C, as shown in Figure 12a,b, and were used to calculate the expected life of two frequently used sections in Spain, by applying the analytical methods. One section specified for heavy traffic, section 031, and the other for light traffic, section 4131. The first section has 30 cm of bituminous mix on a granular base and the second one has 10 cm of bituminous mix also laid on a granular layer, both resting on a subgrade of high bearing capacity.

In the dimensioning of the pavements, the passing of loads is taken into consideration but not the passing of time and the ageing of the bituminous mixes. As has been seen in previous sections, this has a high influence on the response of the bituminous materials and on their failure and fatigue damage process. By applying the usual procedures used in the calculation of pavements, i.e., calculation of stresses starting from a multilayered elastic system and application of the fatigue laws, some authors have analysed the effect that ageing has on the bituminous mixes response. The results of this study are presented in Table 2 for the two aforementioned sections, one for light traffic (less than 8·10^4^ 13-ton axle load applications), with low bituminous mix thickness, and another for heavy traffic (more than 10^7^ 13-ton axle load applications) with high bituminous mix thickness.

These results show that the section for light traffic would fail with very low load applications, less than 6000, shortly after putting it into service if it was located in a cold climate at −5 °C. As the mix ages, this rapid failure situation would now occur at 5 °C. However, at 20 °C, the pavement would withstand more load applications as it aged.

**Figure 13:**
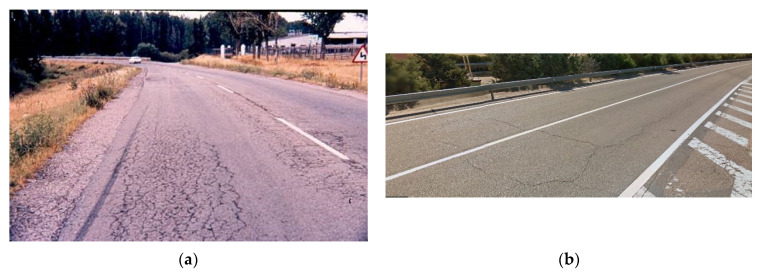
(**a**) Cracks from the top down and (**b**) Typical alligator skin cracking failure.

These types of sections have normally been designed by calculating their fatigue life from the law obtained for the unaged mix at 20 °C. The project period is from 15 to 20 years, which could match the time that elapses from putting the pavement into service up to the ageing of the mix, when a short load application period at low temperatures leads to its rapid fatigue failure (alligator skin cracking, a failure frequently observed in this type of surface, Figure 13a,b).

In the case of pavements with thicker bituminous layers, the application of the same fatigue laws leads to results with a different trend, the colder and more aged the bituminous layer, the greater the number of cycles the pavement will withstand until its fatigue failure. Perhaps this calculation procedure should not be applied to these types of sections, especially considering that the type of failure observed most frequently is not alligator cracking, but longitudinal and transverse cracking. It should also be borne in mind that the mix also loses its ductility due to ageing and the surface ends up cracking transversely. These cracks start on the surface and propagate towards the interior, turning a continuous pavement into a structure composed by slabs. The stresses at the edges of these slabs are higher and this gives rise to their rapid cracking. This is the calculation assumption that has been applied for analysing the sections used in Spain for heavy traffic.

The presence of these transverse and longitudinal cracks has also been highlighted in the LTPP study carried out in the US [24]. This damage is most frequent on pavements located in humid and cold climates.

The design method AASTHO 2002 also analyses the appearance of transverse cracks on the surface of the pavement that propagate towards the interior and quantify and limit this damage [25]. It considers this a surface failure and does not associate it with the structural behaviour of the pavement. This continues to be governed by the failure model based on fatigue cracking, fatigue laws and the sum of damage (Miner’s law). However, as included in the review of the method in 2008, “One reason for the relatively high error terms for both load related fatigue cracking prediction equations (Equations (5)–(7) and Equations (5)–(9)) is that none of the LTPP test sections included in the calibration effort were cored or trenched to confirm whether the fatigue cracks started at the top or bottom of the HMA layers” [26]. Neither was the effect of the rest periods taken into consideration, nor the impact of the cracking on the structural response of the pavement.

As an alternative calculation procedure, it can be assumed that transverse cracking is always going to take place as a consequence of ageing, and it can be proposed to calculate the thickness of the asphalt layers to prevent them from breaking under traffic loads when they reach this condition.

### 7.2. Calculation of the Pavement as Isolated Slabs

The stresses produced at the edge of the slab (*σ_b_*) were calculated by applying Westergaard Equations (10) and (11), which assume an elastic slab resting on an elastic half-space characterised by its subgrade reaction modulus (*k*).

(10) $$\sigma_{b}=\left(\frac{\left(0.803\cdot p\right)}{h^{2}}\right)\cdot \left[4\cdot log\left(\frac{l}{a}\right)+0.282\cdot \left(\frac{a}{l}\right)+0.650\right]$$

(11) $$l={\left[\frac{\left(E\cdot h^{3}\right)}{\left(12\cdot \left(1-\mu^{2}\right)\cdot k\right)}\right]}^{\left(\frac{1}{4}\right)}$$

where

*p*: load (6.5 t)
*h*: thickness
*l*: radius of relative stiffness
*a*: footprint radius (177.06 mm)
*E*: modulus
*µ*: Poisson’s coefficient (0.15)
*k*: subgrade reaction modulus (200 cpi)

**Figure 14:**
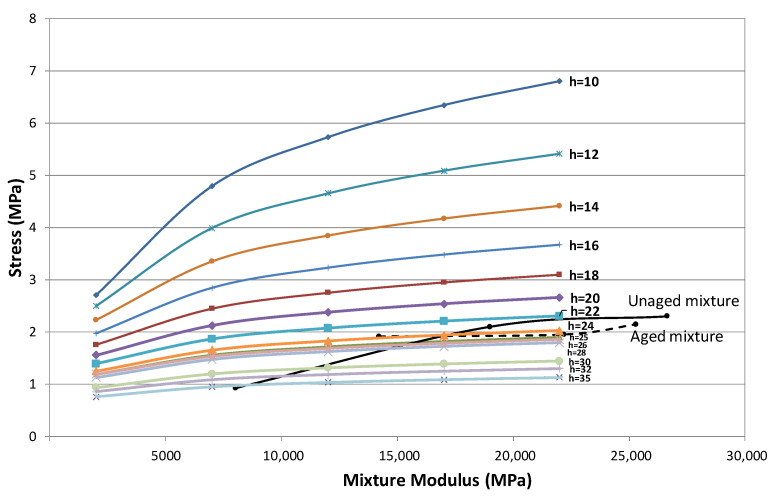
Relationship between the stresses produced in the slab and the resistance of the mixes, for the different slab thicknesses and mixture moduli.

**Figure 15:**
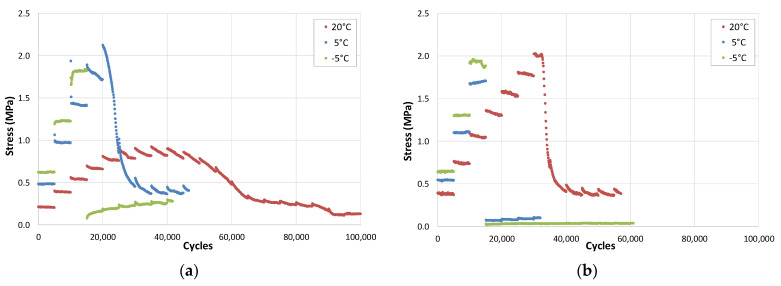
(**a**) Stress evolution for unaged mixtures and (**b**) Stress evolution for aged mixtures, at different test temperatures.

Figure 14 shows the stress curves obtained for a k value of 200 cpi (5.54 kg/cm^3^) corresponding to a subgrade type E3 (CBR > 20) and for different slab thicknesses and layer modulus when a circular load of 6.5 t (13 t standard axle) is applied at the edge of the slab. This figure also shows the curves that relate the initial modulus of the mixture with its maximum resistance obtained from the EBADE test, for both the unaged and aged mixtures, as shown in Figure 15a,b [27].

Figure 14 demonstrates that the stresses that could occur in these slabs would exceed the resistance of the bituminous mixture for thicknesses less than 24–25 cm. This 25 cm thickness, which was used in the construction of the sections for the traffic category between 4 × 10^6^ and 10^7^ 13-ton axle load applications, in the first issue of the Spanish Catalogue of Structural Sections, would probably lead to the breakage of the slabs and to the total ruin of the pavement. These calculations support the decision taken to increase the thickness of the bituminous layers by 5 cm.

Often, when these cracks arrive at the interface of the bituminous layers, they will propagate through the interface, leaving the upper layers detached. These thinner layers break first. One of the most usual maintenance operations is indeed the milling and replacement of these layers. The aforementioned extra thickness added to the pavement may be necessary to safeguard the structural capacity of the pavement, when this damage occurs and the layers are repaired. Considering this damage mechanism, the proposed calculation procedure could be a new tool that could help when applying the analytical methods to the dimensioning of the pavements.

In any event, another important conclusion of the study is the need to use mixes with a ductile response in the temperature range in which they are going to provide service. Moreover, special attention should be paid to the ageing resistance of the bitumens used and to the bonding between layers in the pavement construction, irrespective of the thickness and the method followed in the pavement design. In addition to all these considerations, it is recommended to establish a closer link with the real performance conditions of the road and use advanced techniques to measure the different distresses [28,29].

## 8. Conclusions

The analysis of the response of the bituminous materials, both mixes and bituminous binders, under the application of cyclic loading, with the introduction of different strain, temperature, and rest period levels, through the use of the EBADE test, offers a broader perspective on the behaviour of these materials which should be taken into consideration in the application of the pavement dimensioning calculation models.

In the cyclic tests, the bituminous materials pass from ductile to brittle behaviour, depending on their temperature and state of ageing.

The behaviour of these materials in the cyclic loading tests at the temperature at which they have a ductile and viscous response is associated with a thixotropic model. Their response is characterized by:

- There are not different phases in the cyclic fatigue tests, but a continuous change of the rheological characteristics depending on the history of the applied stresses.
- Depending on the strain level imposed, the mixture may recover its initial properties if an unloading process is applied or the test is stopped. This recovery is characteristic of the materials with a thixotropic response. This is not a consequence of self-healing.
- The rest periods can significantly increase the number of applications before reaching the failure for any strain level.
- The way the same load is applied affects the results. Miner’s law is not met.

When the temperature goes down, bituminous materials adopt a response more similar to that of elastic, stiff, and brittle materials. As for the effect of aging:

- The brittle response of the mix takes place at higher temperatures, around 10 °C higher than that of the original mix.

The analytical pavement dimensioning methods should take into consideration both the effect that the rest periods have and the evolution of the response of the bituminous mixes with ageing.

- Moreover, the transverse cracking of the pavement should be introduced into the pavement calculation.

## Figures and Tables

**Table 1:** Properties of 13/22 and 60/70 bitumens.

Parameter	Unit	Test Method	13/22 *	60/70 *
Penetration at 25 °C	0.1 mm	EN 1426	17	64
Softening point	°C	EN 1427	67.3	51.7
Fraass breaking point	°C	EN 12593	−5	−17
After RTFOT				
Mass variation	%	EN 12607-1	0.35	0.5
Retained pen	%	EN 1426	10	32
Softening point increment	°C	EN 1427	7.5	9.6

*: the penetration ranges of bitumens predate the European harmonization process (60/70 is current 50/70).

**Table 2:** Horizontal strains (ε) and fatigue lives (N) calculated from the estimated fatigue laws.

Bitumen Conditioning	Temp. (°C)	Modulus (MPa)	Section 031		Section 4131	
			ε	N	ε	N
**Unaged**	−5	26,645	21.8	7.72 × 10^+12^	89.5	5682
	5	18,983	28.7	2.79 × 10^+10^	113	45,831
	20	8013	57.1	6.42 × 10^+9^	194	154,197
**Aged**	−5	24,992	23	1.53 × 10^+15^	93.5	128
	5	21,769	25.7	1.25 × 10^+15^	103	326
	20	14,192	36.3	3.13 × 10^+22^	136	556,880

## Data Availability

Data is contained within the article.
